# Correlated changes in stress resistance and biochemical parameters in response to long-term protein restriction in *Drosophila melanogaster*

**DOI:** 10.1098/rsos.231741

**Published:** 2024-06-05

**Authors:** Sudhakar Krittika, Pankaj Yadav

**Affiliations:** Fly Laboratory # 210, Anusandhan Kendra-II, School of Chemical & Biotechnology, SASTRA Deemed to be University, Thanjavur, Tamil Nadu 613401, India

**Keywords:** protein restriction, stress resistance, biochemical parameters, triglyceride, glucose

## Abstract

Studies in fruit flies, *Drosophila melanogaster*, have observed considerable variation in the effect of dietary protein restriction (PR) on various fitness traits. In addition, not only are there inconsistent results relating lifespan to stress resistance, but also the long-term effects of PR are unexplored. We study PR implementation across generations (long term) hypothesizing that it will be beneficial for fitness traits, stress resistance and storage reserves due to nutritional plasticity transferred by parents to offspring in earlier *Drosophila* studies. By imposing two concentrations of PR diets (50% and 70% of control protein) from the pre-adult and adult (age 1 day) stages of the flies, we assessed the stage-specific and long-term effect of the imposed PR. All long-term PR flies showed increased resistance against the tested stressors (starvation, desiccation, H_2_O_2_-induced oxidative stress). In addition, we also found long-term PR-induced increased stress resistance across generations. The PR flies also possessed higher protein and triglyceride (TG) content, reduced glucose and unaffected glycogen levels. We also assayed the effect of returning the PR flies to control (AL) food for a single generation and assessed their biochemical parameters to witness the transient PR effect. It was seen that TG content upon reversal was similar to AL flies except for PRI70 males; however, the glucose levels of PR males increased, while they were consistently lower in females. Taken altogether, our study suggests that long-term PR implementation contributes to increased stress resistance and was found to influence storage reserves in *D. melanogaster*.

## 1. Introduction

Living organisms depend on food and nutrient sources to develop, survive and maintain normal body function. Once the food supply is in excess, the body undertakes metabolic modifications to store the nutrients for either periods of starvation or other energy-consuming processes [1–4]. It is also notable that according to the resource reallocation hypothesis, nature may favour organisms that could establish phenotypic plasticity [5,6] and there is an ideal balance when allocating resources between reproduction and maintenance including increased stress resistance [7]. Interestingly, when there is a deficit of nutrients in the environment or laboratory-imposed diet restriction (DR; henceforth), it is reported that the storage reserves of organisms including fruit fly *Drosophila melanogaster* are increased [8,9], thereby allowing them to sustain brief periods of starvation. Protein restriction (PR; henceforth) is chosen because of the effects credited to amino acids and P:C ratios rather than the restriction of carbohydrates or total calories [10,11]. Prior research indicates that the developmental diet might influence lifespan while some studies suggest that reducing yeast during the developmental stage and early age can extend lifespan [12,13]. It is also reported that the changes in food composition and thereby the nutrients like protein, carbohydrates and fats in the food are key factors that affect the starvation resistance (SR; henceforth) of the flies [14–16]. Since it has been well reported that the nutrition and fitness of the parents are capable of influencing the offspring's fitness, studying the effect of DR across generations is crucial. Inter-generational studies have shown that high sugar parental diet affects offspring with obese-like phenotype, but not lifespan [17,18]. Interestingly in *C. elegans*, the effect of SR was shown to influence the future generations wherein F_1_ and F_2_ generations of starved F_0_ parents were more resistant to starvation [19]. The SR in flies is known to have ecological significance because of the uncertainty in food availability in the fluctuating natural habitat [20].

Starved conditions or nutritional stress in larvae may trigger behavioural changes and also influence their food choices [21]. One such behaviour is cannibalism which is displayed by *Drosophila* larvae under a nutritionally deprived environment, wherein they feed on conspecifics and also other species; however, there was no evidence that such behaviour has any benefit for the fly in terms of lifespan extension [21–23]. Some studies show that oxidative stress can reduce the lifespan of flies [24–26], while others show that starvation and oxidative stress resistance might not be tightly linked to longevity [27–29]. Therefore, this challenges the free radical hypothesis of ageing which states that the gradual accumulation of oxidative damage in the cells is one of the nine suggested hallmarks of ageing [30]; although, this is beyond the scope of the current study.

SR is a highly variable and heritable trait in flies, and its correlated response in terms of development and lifespan has been seen in populations selected for increased SR [31–35]. In periods of food deprivation, sugars and triglycerides (TGs) are depleted and restored upon refeeding. To ensure organismal survival during starvation, regulatory mechanisms control the levels of energy reserves such as lipids or TGs, decrease metabolite utilization rates, or reduce the need for energy-related metabolites [36–38]. However, there is evidence both for [38,39] and against [40,41] the idea that storage lipids levels might correlate positively with SR in *D. melanogaster*. Moreover, the storage levels of other reserves like glycogen are also subjected to alterations under nutritionally challenged environments [42,43]. The existence of inter- and trans-generational effects spanning three generations on offspring TG levels was reported to be dependent on the sex and origin of the parent [44]. Hence, as discussed earlier and in the view of understanding how parental diet affects phenotypes and storage in offspring, the trans-generational effect on the variations in SR and its mediated influence on storage reserves will be an important addition to the *Drosophila* nutrition studies.

The results of the current study report, for the first time, that PR may have long-term effects (across generations) on resistance to various stressors such as starvation, desiccation, oxidative stress along with the levels of storage reserves like TG, glycogen, protein and glucose content. Our results revealed that PR flies showed increased starvation and desiccation resistance (SDR), and oxidative stress resistance compared to that of AL (ad libitum; henceforth) flies. We also see that free glucose levels (circulating glucose in the body fluids) in Assay 2 flies were variable and possibly require more generations or a better assessment for a conclusion. Overall, our study confirms the trans-generational and transient effect of PR on specific parameters and stages of PR implementation, thereby supplementing studies that aim to understand the holistic effect of PR diets in *D. melanogaster*.

## 2. Material and methods

### 2.1. Fly stock maintenance and fly culture

Wild-type *D. melanogaster* (*Canton S*-CS) were maintained on the respective protein-restricted diets (50% and 70%; selected based on our earlier works in [13]; S Krittika & P Yadav, unpublished data, 2024) alongside control food (AL) in a plexiglass cage of length-25 cm, breadth-20 cm and height-15 cm under a constant temperature of 25 °C (±0.5 °C) and humidity (70 ± 5%). Here, the two types of fly maintenance protocols were followed, first named Assay-1 in which the PR diets (50% and 70% protein restricted when compared with that of AL control which is considered as 100% protein diet) were subjected to flies from their egg stage. The second protocol is named Assay 2, wherein the PR50% and PR70% were subjected to flies from their adult stage (eclosion Day 1). During egg collection for the next generation, eggs from Assay 1 PR-fed flies were collected and transferred to the vials containing respective PR diets of 50% and 70%, while for AL and Assay 2-PR flies, the egg collection was done in AL food. This was done to understand the effect of lifelong PR (Assay 1) and in adulthood (Assay 2) only and the various parameters have been tested across generations varying between gen 1, 2, 15, 30, 45 and reversal at 48. Immediately after eclosion, the control AL flies (AL food-fed flies, henceforth) and the PR flies were transferred to the cages where the respective diets of AL and PR were given to the AL and PR flies in both Assay 1 and 2 flies. We also did experiments on PR flies with reversal at 48 generations by standardizing the stock, and by maintaining the flies for one generation in AL food to avoid the non-genetic parental effect. The agar from HIMEDIA and instant dry yeast from Gloripan were used for fly food preparation media. The compositions of the control and the PR diets are reported elsewhere [13].

### 2.2. Starvation resistance assay

On the 12-day age of adult flies, egg collection for the experiments was done from population cages maintained on the respective AL and PR diets into their corresponding food vials. Approximately 40 eggs laid in 3 h were collected in each vial, such as to get the desired number of flies for the experiment. Freshly eclosed virgin flies were separated using mild CO_2_ and were transferred into the vials with 4 ml of solidified agar media. The set-up of ten such vials containing 10 flies each was followed for different diets and sex. We then measured the lifespan of every fly until the death of the last fly in each vial. Similarly, the grand average of lifespan over the 10 replicate vials refers to the average lifespan of a particular treatment, say AL or PR50 or PR70%. The vials were maintained at a temperature of approximately 25 °C, approximately 70% humidity and under LD12:12 regimes (light: dark 12:12 hr cycles) and were checked for the death of flies every 2 h regularly until the death of the last fly in each vial.

### 2.3. Starvation and desiccation resistance assay

The experimental set-up and procedure were the same as that of the above-mentioned SR assay protocol, with the only difference being that, instead of vials with agar, heat-dried empty vials were used. This ensures that the flies are starved and are not exposed to humidity from the food.

### 2.4. Oxidative stress resistance assay

One of the other common stressors other than starvation in nutrition studies is oxidative stress. The experiment was carried out by subjecting 10-day-old virgin males and females to vials containing food with H_2_O_2_ (30% w/v hydrogen peroxide; spectrum reagents and chemicals) solution. The protocol was adapted from the study reported elsewhere with slight modifications like the fly number and adding H_2_O_2_ to the food [45,46]. One per cent H_2_O_2_ was added to the fly media and the vial was cotton-plugged after the media solidified. For the experiment, each vial housed 10 flies (tested on both males and females separately) and 10 vials for each experimental set-up were used (10 flies × 10 vials/ diet/ sex). The collected AL and PR flies were put in normal control fly food (experimental control) and H_2_O_2_-added food vials, and checked for the death of flies every 2 h, which was continued until the death of the last fly in each vial (approx. 3–4 days). The stress resistance was calculated as the average number of hours of survival post-transfer into the H_2_O_2_ food vials.

### 2.5. Protein content estimation

Long-term PR-imposed flies were transferred to AL food at generation 48 (indicated as R, to assess the plasticity of the PR effect across generations), irrespective of PR concentration or assay background. The long-term PR (PR over multiple generations) and R48 flies were tested for their biochemical parameters, protein, TG, free glucose and glycogen content.

The protein estimation was done using a Bradford kit from Himedia (ML178-1PK). Virgin flies were separated as males and females using mild CO_2_ and five flies of each sex and diet were washed with 1 ml PBS (Phosphate buffer saline; Himedia; TL1031) and the flies were put in a microfuge tube. One hundred and one millilitres of ice-cold PBST (Phosphate buffer saline tween) was added and then homogenized using a pellet pestle (Sigma; Z359955-1EA). The samples were centrifuged at 12 000 r.p.m. for 10 min at 4 °C. Further, 5 µl of the fly sample or the prepared standard stock solution (Himedia; MBT129; as given in the protocol of Bradford assay kit, Himedia) were added as triplicates in 96-well plate, and 5 µl of PBST was added as blank in the wells. Following this, 250 µl of Bradford reagent (Himedia; ML106) was added to all the wells including blank, and the 96-well plate was kept on the shaker for 30 secs. The plate was incubated at 28 °C for 30 min and the absorbance was measured at 595 nm. The values were normalized with the body weight of the flies used and expressed as estimated protein (mg)/ body weight (mg).

### 2.6. Quantification of triglycerides

The TG quantification was done with slight modifications (like fly sample number) from the protocol of a study reported elsewhere [47]. Briefly, five newly eclosed adult flies (males and females each) were washed and homogenized using a pellet pestle as described earlier. The sample was centrifuged and the supernatant was heated for 10 min at 70 °C. Twenty millilitres of either the glycerol standard or the fly sample were added to two microfuge tubes. Further, 20 µl of TG reagent (Triglyceride reagent; Sigma; T2449) was added in one of the tubes with the standard or sample to measure the TG content, while in another tube 20 µl of PBST was added to measure the total free glycerol present. The tubes were incubated for 45 min at 37 °C, then centrifuged at 8000 r.p.m. for 3 min, and 30 µl of each sample was added to a 96-well plate. Free glycerol reagent (100 µl; Sigma-F6428) was added to all tubes and mixed well. The plates were covered with parafilm to prevent evaporation and incubated for 5 min at 37 °C. After incubation, the plates were centrifuged and the absorbance was measured at 540 nm in a microplate reader.

### 2.7. Quantification of free glucose and glycogen

Glucose content in the fly body is a source of immediate energy, and changes in its level are not expected under PR. To test this hypothesis, free glucose and glycogen quantification was done using an HK kit (Sigma; GAHK20-1KT) with the procedure as described [47]. The washing and homogenization step followed the method described earlier using freshly eclosed flies (5 flies of each sex and diet). The supernatant was heated for 10 min at 70 °C and later centrifuged in a pre-chilled centrifuge at 4 °C. The 20 µl of heat-treated samples were diluted with PBS in a 1 : 3 ratio, and 20 µl of the diluted sample was then added to one tube containing amyloglucosidase solution (to measure glycogen and glucose) and 20 µl in a tube containing PBS (untreated; to measure glucose). Further, 30 µl of diluted sample from each of the tubes is added to the 96-well plate, sealed with parafilm and incubated for 60 min at 37 °C. The plates were then spun for approximately 30 s, and 100 µl of Hexokinase reagent was added to each well, following which the plates were again incubated at RT for 15 min. The absorbance of the plate is measured at 340 nm and the glycogen levels are obtained by subtracting the free glucose (untreated samples) from the amyloglucosidase-treated sample.

### 2.8. Statistical analyses

All statistical analyses were done using analysis of variance (ANOVA), followed by Tukey's honestly significant difference (HSD) test for multiple comparisons on STATISTICA for Windows Release 7 (StatSoft Inc. 1995, 2004) and GraphPad Prism version 5.00 for Windows. The statistical significance is considered if *p* < 0.05 and error bars are plotted with mean ± s.d.

## 3. Results

**Figure 1.**
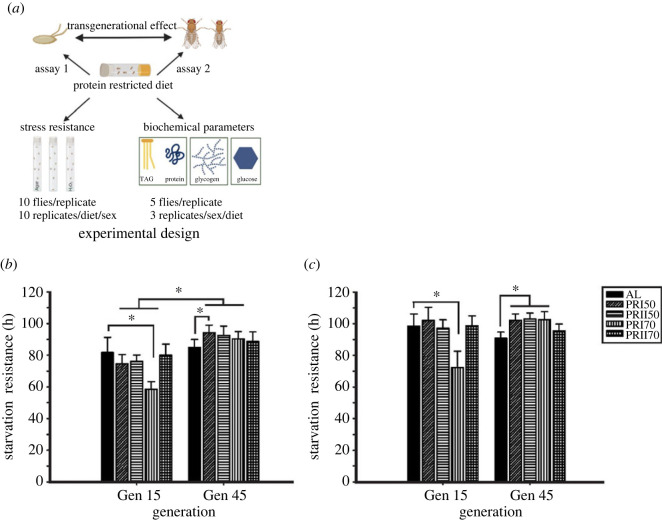
Long-term PR implementation effect on starvation resistance: The assayed parameters and their experimental design are represented (*a*). The starvation resistance of males (*b*) and females (*c*) shows increased resistance to starvation in PR-imposed flies. The statistical significance is considered if *p* < 0.05 indicated by * and error bars are plotted with mean ± SD. The lines over the result bars are drawn based on significant comparisons between control and PR diets, as well as generations.

The experimental design of the tested parameters is given in figure 1 *a*.

### 3.1. Starvation resistance

In male flies, there was a significant effect of diet (D; *F*_4,83_ = 10.20, *p <* 0.0001), generation (G; *F*_1,83_ = 157.55, *p <* 0.0001) and their interaction (D × G; *F*_4,83_ = 16.45, *p <* 0.0001; figure 1 *b*; table 1) on the survival of flies under starvation. Tukey's test shows that the SR (quantified by hours of survival) of PRI70 (70% Protein diet from the pre-adult stage- Assay 1) males were lower at gen 15 when compared with AL, while at gen 45, it was close to AL. Similarly, the SR of PRI50 flies (flies subjected with PR50% from pre-adult stage-Assay 1) was greater at gen 45 when compared with gen 15. In females, ANOVA followed by post hoc multiple comparisons on SR data revealed a significant effect of diet (*F*_4,83_ = 15.26, *p <* 0.0001), generation (*F*_1,83_ = 14.27, *p <* 0.0003) and their interaction (*F*_4,83_ = 27.29, *p <* 0.0001; figure 1 *c* and table 1). The SR of PRI50, PRII50 (50% Protein diet from Day 1 of adult stage- Assay 2) and PRI70 females at gen 45 is higher than that of AL. Taken together, these data show that all PR yield flies with similar or increased SR to AL at gen 45, showing that long-term PR probably increases their resistance as a response to the trans-generational effect.

**Table 1.** ANOVA details of starvation resistance (SR) and starvation and desiccation resistance (SDR) under a long-term imposed PR diet.

assay	effect	d.f.	MS effect	d.f. error	MS error	*F*	*p*-value <
SR							
males	diet (D)	4	365.9	83	35.9	10.20	0.0001
	generation (G)	1	5653.5	83	35.9	157.55	0.0001
	diet × generation (D × G)	4	590.1	83	35.9	16.45	0.0001
females	diet (D)	4	618.8	83	40.5	15.26	0.0001
	generation (G)	1	578.5	83	40.5	14.27	0.0003
	D × G	4	1106.3	83	40.5	27.29	0.0001
SDR							
males	diet (D)	4	358.3	84	17.41	20.58	0.0001
	generation (G)	1	944.9	84	17.41	54.26	0.0001
	D × G	4	268.1	84	17.41	15.4	0.0001
females	diet (D)	4	228.7	84	18.4	12.44	0.0001
	generation (G)	1	130.7	84	18.4	7.11	0.0092
	D × G	4	205.9	84	18.4	11.19	0.0001

### 3.2. Starvation and desiccation resistance

**Figure 2.**
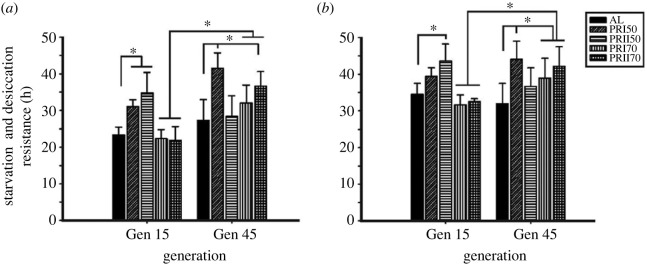
Long-term PR implementation effect on starvation and desiccation resistance: PR imposed males (*a*) and females (*b*) show increased resistance to starvation and desiccation. Other details are the same as mentioned in figure 1.

SDR of males showed a significant effect of diet (*F*_4,84_ = 20.58, *p <* 0.0001), generation (*F* _1,84_ = 54.26, *p <* 0.0001) and their interaction (*F*_4,84_ = 15.4, *p <* 0.0001; figure 2 *a* and table 1). At gen 1, PRI50 and PRII50 males had higher SDR than AL, while at gen 45, PRI50 and PRII70 (70% Protein diet from the pre-adult stage- Assay 2) showed higher SDR. Similarly, the females showed a statistically significant effect of diet (*F*_4,84_ = 12.44, *p <* 0.0001), generation (*F*_1,84_ = 7.11, *p <* 0.0092) and their interaction (*F*_4,84_ = 11.19, *p <* 0.0001; figure 2 *b*; table 1). The SDR of PRII50 females is greater than that of AL at gen 15, while the pattern of higher SDR of PR females at gen 45 is similar to that of males. Thus, these results suggest that the PR had benefitted the flies with higher SDR than AL and between the tested generations.

### 3.3. Oxidative stress resistance

**Figure 3.**
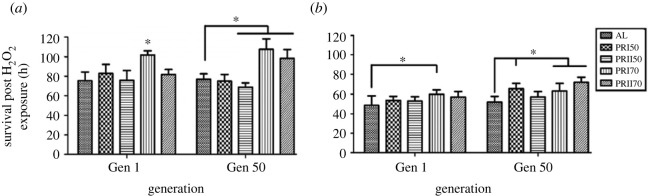
Long-term PR implementation effect on oxidative stress resistance: the resistance to oxidative stress induced by H_2_O_2_ in males (*a*) and females (*b*) at gen 1 versus gen 50 shows increased oxidative stress resistance under certain PR diets. Other details are the same as mentioned in figure 1.

There was a significant effect of diet (*F*_4,90_ = 57.09, *p <* 0.0001) and interaction with generation (*F*_4,90_ = 8.37, *p <* 0.0001), but not of generation (*F*_1,90_ = 1.32, *p <* 0.2529; figure 3 *a* and table 2) on the oxidative stress resistance of flies. At gen 1, PRI70 males have higher oxidative stress resistance; while at gen 50, PRII50, PRI70 and PRII70 males show greater stress resistance than AL (electronic supplementary material, figure S1), showing long-term PR can alter this resistance. Multiple comparisons on the oxidative stress resistance data of females revealed a statistically significant effect of diet (*F*_4,90_ = 17.17, *p <* 0.0001), generation (*F*_1,90_ = 38.70, *p <* 0.0001) and their interaction (*F*_4,90_ = 4.03, *p <* 0.0047; figure 3 *b* and table 2). Similar to males, PRI70 females have higher oxidative stress resistance at gen 1; while at gen 50, PRI50, PRI70 and PRII70, the females show greater stress resistance than AL (electronic supplementary material, figure S1). Thereby, the results show that the PRI70 males and females in gen 1 have higher resistance, and flies increase their resistance to H_2_O_2_-induced oxidative stress upon single (gen 1) and multiple (gen 50) generations of PR diet.

**Table 2.** ANOVA details of oxidative stress resistance under long-term imposed PR diet.

assay	effect	d.f.	MS effect	d.f. error	MS error	*F*	*p*-value <
males	diet (D)	4	3447.3	90	60.4	57.09	0.0001
	generation (G)	1	79.9	90	60.4	1.32	0.2529
	diet × generation (D × G)	4	505.6	90	60.4	8.37	0.0001
females	diet (D)	4	619.8	90	36.1	17.17	0.0001
	generation (G)	1	1397.3	90	36.1	38.70	0.0001
	D × G	4	145.6	90	36.1	4.03	0.0047

### 3.4. Protein content

**Figure 4.**
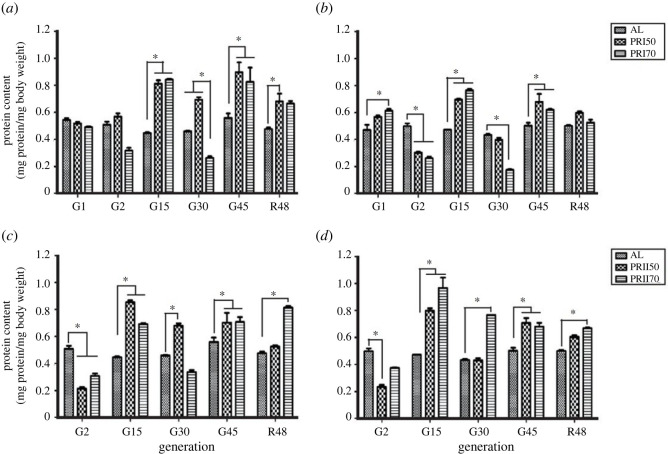
Total protein content in AL and PR flies: the protein content of Assay 1-males (*a*), females (*b*) and Assay 2-males (*c*), females (*d*) is significantly different under long-term PR implementation. Other details are the same as mentioned in figure 1.

Protein content of Assay 1-males showed significant effect of diet (*F*_2,36_ = 43.44, *p <* 0.0001), generation (*F*_5,36_ = 33.7, *p <* 0.0001) and their interaction (*F*_10,36_ = 14.45, *p <* 0.0001; figure 4 *a*; electronic supplementary material, table S1). PR flies at gen 1 and 2 had similar protein content (similar to AL) which increased in further generations particularly the PRI50 male flies upon reversal (R48; figure 4 *a*; electronic supplementary material, table S1). It is also seen that upon reversal to AL for a single generation, the PR content of PRI50 flies is significantly lower than their counterparts at gen 45. In the case of females, ANOVA showed significant effect of diet (*F*_2,36_ = 13.99, *p <* 0.0001), generation (*F*_5,36_ = 119.85, *p <* 0.0001) and their interaction (*F*_10,36_ = 33.01, *p <* 0.0001; figure 4 *b*; electronic supplementary material, table S1). *Post hoc* multiple comparisons by Tukey's test revealed that PRI70 flies at gen 1 had higher protein content which was reduced at gen 2. Moreover, across generations, we report that the protein content in PR flies is more than that of AL (at gen 45), but is similar to AL at R48 (figure 4 *b*; electronic supplementary material, table S1). Taken together, the effect of PR on Assay 1 flies shows that PRI70 had higher protein content at gen 1 and the reversal has an effect in mediating the protein content at gen 45 against long-term PR implementation.

Assay 2-males showed significant effect of diet (*F*_2,30_ = 23.68, *p <* 0.0001), generation (*F*_4,30_ = 86.44, *p <* 0.0001) and their interaction (*F*_8,30_ = 46.97, *p <* 0.0001; figure 4 *c*; electronic supplementary material, table S1). *Post hoc* multiple comparisons by Tukey's test revealed that at gen 2, the protein content of PR males was lower than AL which increased in further generations and is consistent under reversal as well as in PRII70 males. ANOVA on total protein content data of females revealed significant effect of diet (*F*_2,30_ = 84.96, *p <* 0.0001), generation (*F*_4,30_ = 84.99, *p <* 0.0001) and their interaction (*F*_8,30_ = 30.17, *p <* 0.0001; figure 4 *d*; electronic supplementary material, table S1). Multiple comparisons by Tukey's test suggest that Assay 2 females showed lower protein content in PRII50 flies at gen 1 alone; across generations, they exhibited an increased protein content including reversal generation. Thereby, it is seen that under Assay 2, the protein content of males and females showed a similar response of PR upon long-term implementation. Thus, taken together, we conclude that there exist stage-specific differences in protein content upon PR implementation and that the reversal flies exhibit similar and increased protein content.

### 3.5. Triglyceride content

**Figure 5.**
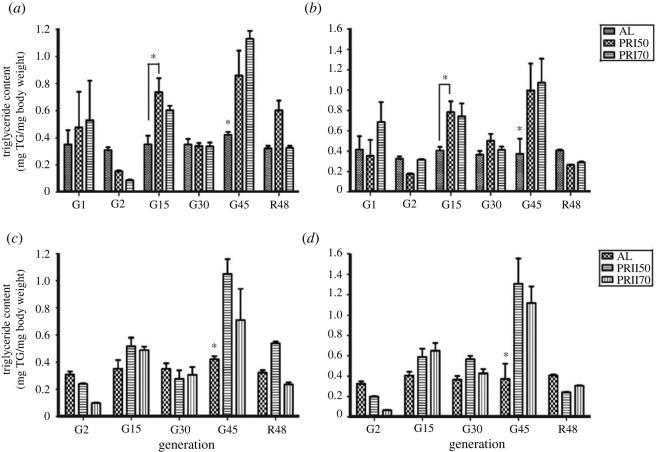
Triglyceride content in AL and PR flies: the triglyceride content of Assay 1-males (*a*), females (*b*) and Assay 2-males (*c*), females (*d*) are significantly higher under long-term PR implementation and drops drastically to the TG content similar to AL upon reversal (R48). Other details are the same as mentioned in figure 1.

ANOVA on TG content data of Assay 1-males showed a significant effect of diet (*F*_2,36_ = 12.83, *p <* 0.0001), generation (*F*_5,36_ = 31.5, *p <* 0.0001) and their interaction (*F*_10,36_ = 7.51, *p <* 0.0001; figure 5 *a* and table 3). Multiple comparisons using Tukey's test revealed that the PRI50 flies at gen 15 showed higher TG content, while at gen 45, both PRI50 and PRI70 males showed higher TG content. Surprisingly, upon reversal, the TG content of PR males was similar to that of AL, while PRI70 flies upon reversal had lower TG content when compared with their counterparts at gen 45. In the case of females, ANOVA revealed a significant effect of diet (*F*_2,36_ = 13.10, *p <* 0.0001), generation (*F*_5,36_ = 25.68, *p <* 0.0001) and their interaction (*F*_10,36_ = 7.13, *p <* 0.0001; figure 5 *b*; table 3). The TG content of PR females at R48 was similar to that of AL. Interestingly, both PRI50 and PRI70 flies at R48 had lower TG content when compared with that at gen 45. Thus, these results suggest that the effect of PR is mostly an environmental transient effect, which upon reversal to AL food decreases the storage reserves similar to that of AL.

**Table 3.** ANOVA details of triglyceride content under long-term PR diet implementation.

assay	effect	d.f.	MS effect	d.f. error	MS error	*F*	*p*-value <
Assay 1							
males	diet (D)	2	0.1626	36	0.013	12.83	0.0001
	generation (G)	5	0.3992	36	0.013	31.5	0.0001
	diet × generation (D × G)	10	0.0951	36	0.013	7.51	0.0001
females	diet (D)	2	0.1923	36	0.015	13.10	0.0001
	generation (G)	5	0.3770	36	0.015	25.68	0.0001
	D × G	10	0.1047	36	0.015	7.13	0.0001
Assay 2							
males	diet (D)	2	0.1371	30	0.005	24.26	0.0001
	generation (G)	4	0.3415	30	0.005	60.42	0.0001
	diet × generation (D × G)	8	0.0737	30	0.005	13.04	0.0001
females	diet (D)	2	0.1634	30	0.008	19.17	0.0001
	generation (G)	4	0.7121	30	0.008	83.53	0.0001
	D × G	8	0.1792	30	0.008	21.02	0.0001

ANOVA on Assay 2-male data showed a significant effect of diet (*F*_2,30_ = 24.26, *p <* 0.0001), generation (*F*_4,30_ = 60.42, *p <* 0.0001) and their interaction (*F*_8,30_ = 13.04, *p <* 0.0001; figure 5 *c* and table 3). Similarly, in the case of females, ANOVA revealed the significant effect of diet (*F*_2,30_ = 19.17, *p <* 0.0001), generation (*F*_4,30_ = 83.53, *p <* 0.0001) and their interaction (*F*_8,30_ = 21.02, *p <* 0.0001; figure 5 *d* and table 3). Multiple comparisons using Tukey's test revealed that the TG content of Assay 2 PR flies is much like that of AL in any of the tested generations including reversal, except for higher TG content at gen 45. Thus, these results suggest that long-term PR implementation had increased the TG content of Assay 2 PR flies which becomes similar to AL at R48. Therefore, interestingly, the reversal of PR flies to AL for a single generation can nullify the observed increased TG levels.

### 3.6. Free glucose content

**Figure 6.**
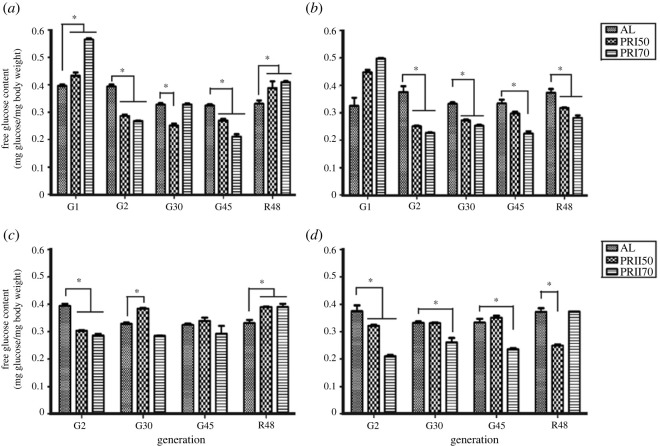
Free glucose content in AL and PR flies: the free glucose content of Assay 1-males (*a*), females (*b*) and Assay 2-males (*c*), females (*d*) is significantly lower under long-term PR implementation and increases upon a reversal in males but not in females. Other details are the same as mentioned in figure 1.

Assay 1-males showed a significant effect of diet (*F*_2,20_ = 19.90, *p <* 0.0001), generation (*F*_4,20_ = 159.1, *p <* 0.0001) and their interaction (*F*_8,20_ = 60.58, *p <* 0.0001; figure 6 *a*; electronic supplementary material, table S2). At gen 1, the PR male flies showed higher free glucose levels (when compared with AL) which were also increased when compared with that at other tested generations (2, 30 and 45), but similar to R48. In the case of females, there was a significant effect of diet (*F*_2,20_ = 35.80, *p <* 0.0001), generation (*F*_4,20_ = 48.48, *p <* 0.0001) and their interaction (*F*_8,20_ = 48.93, *p <* 0.0001; figure 6 *b*; electronic supplementary material table S2). Multiple comparisons by Tukey's test showed that the free glucose levels of PR flies are similar to that in AL at gen 1 but lower in all other tested generations. Moreover, in the reversal generation, the glucose levels of the PR flies increased in males, while it was consistently lower in females. Thus, the long-term PR implementation lowers free glucose content which can be reversed in males, but not in females at least until 48 generations.

ANOVA followed by multiple comparisons by Tukey's test on the free glucose content of Assay 2-males revealed the significant effect of diet (*F_2,16_* = 13.07, *p <* 0.0004), generation (*F_3,16_* = 20.03, *p <* 0.0004) and their interaction (*F_6,16_* = 13.81, *p <* 0.0001; figure 6 *c*; electronic supplementary material, table S2). The free glucose content of PR flies (PRII50 and PRII70) was lower when compared with that of AL flies at gen 2 and R48; while PRII50 males had higher glucose content at gen 30, and similar to AL at gen 45. In the case of females, there was a significant effect of diet (*F_2,16_* = 91.47, *p <* 0.0001) and their interaction (*F_6,16_* = 47.35, *p <* 0.0001), but not of generation (*F_3,16_* = 3.16, *p <* 0.0859; figure 6 *d*; electronic supplementary material, table S2). Both PRII50 and PRII70 females at gen 2 showed decreased glucose content when compared with AL. However, only PRII70 females showed lower glucose content at gen 30 and 45 than that of AL. Interestingly, upon reversal of PR flies to AL, PRII50 females showed lower glucose content which was otherwise similar to AL in the earlier tested generations. Thus, taken together, these results suggest that the glucose content in Assay 2 flies is variable and it requires more generations to interpret the PR effect on the glucose content.

### 3.7. Glycogen content

**Figure 7.**
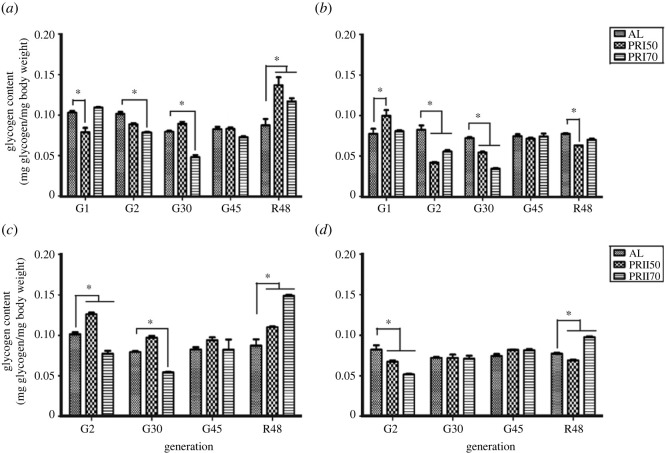
Glycogen content in AL and PR flies: the glycogen content of Assay 1-males (*a*), females (*b*) and Assay 2-males (*c*), females (*d*) upon long-term PR implementation is much like AL and increases upon reversal to AL in males. Other details are the same as mentioned in figure 1.

ANOVA on the glycogen content of Assay 1-PR males showed a significant effect of diet (*F*_2,20_ = 7.31, *p <* 0.0041), generation (*F*_4,20_ = 69.17, *p <* 0.0001) and their interaction (*F*_8,20_ = 18.81, *p <* 0.0001; figure 7 *a* and table 4). Multiple comparisons by Tukey's test suggest that PRI50 males (at gen 1) and PRI70 males (at gen 2 and 30) showed lower glycogen content when compared with AL, while at the latest tested gen 45, the glycogen content of PR males was much like that of AL flies. Upon reversal to AL, it was seen that the glycogen content of PR flies increases at R48. ANOVA followed by multiple comparisons by Tukey's test on the females' glycogen content data showed a significant effect of diet (*F*_2,20_ = 34.77, *p <* 0.0001), generation (*F*_4,20_ = 30.81, *p <* 0.0001) and their interaction (*F*_8,20_ = 23.82, *p <* 0.0001; figure 7 *b* and table 4). PRI50 females showed increased glycogen content at gen 1, while both PRI50 and PRI70 females exhibited lower glycogen content at gen 2 and 30. The glycogen content of PR females at gen 45 was similar to that of AL, but upon reversal, PRI50 females exhibited lower glycogen content. Thus, the results of this study show that upon long-term PR implementation, the females had adapted to glycogen content like AL, which was decreased upon reversal to AL and is, thereby, opposite to the reduced glycogen reserves witnessed in males upon PR implementation.

**Table 4.** ANOVA details of glycogen content under long-term PR diet implementation.

assay	effect	d.f.	MS effect	d.f. error	MS error	*F*	*p*-value <
Assay 1							
males	diet (D)	2	0.0003	20	0.00006	7.31	0.0041
	generation (G)	4	0.0023	20	0.00006	69.17	0.0001
	D × G	8	0.0010	20	0.00006	18.81	0.0001
females	diet (D)	2	0.0008	20	0.00002	34.77	0.0001
	generation (G)	4	0.0014	20	0.00002	30.81	0.0001
	D × G	8	0.0005	20	0.00002	23.82	0.0001
Assay 2							
males	diet (D)	2	0.0013	16	0.00007	17.10	0.00
	generation (G)	3	0.0026	16	0.00007	45.31	0.0001
	D × G	6	0.0016	16	0.00007	22.06	0.0001
females	diet (D)	2	0.0001	16	0.00001	3.95	0.0404
	generation (G)	3	0.0004	16	0.00001	12.48	0.0022
	D × G	6	0.0005	16	0.00001	36.96	0.0001

ANOVA followed by post hoc multiple comparisons by Tukey's test on Assay 2-males showed significant effect of diet (*F*_2,16_ = 17.10, *p <* 0.0001), generation (*F*_3,16_ = 45.31, *p <* 0.0001) and their interaction (*F*_6,16_ = 22.06, *p <* 0.0001; figure 7 *c* and table 4). PRI50 males yielded higher glycogen content at gen 2, and PRII70 flies showed lower glycogen at gen 2 and 30. However, at R48, it was seen that the PR males exhibited increased glycogen content, again revealing that the long-term PR lowered glycogen content in the earlier generations. In the case of females, ANOVA revealed a statistically significant effect of diet (*F*_2,16_ = 3.95, *p <* 0.0404), generation (*F*_3,16_ = 12.48, *p <* 0.0022) and their interaction (*F*_6,16_ = 36.96, *p <* 0.0001; figure 7 *d* and table 4). It was seen that the PR females in gen 2 had lower glycogen content than that of AL females, while in the later tested generations, it was similar to AL flies. Interestingly, at R48 PRII50 has lower and PRII70 has higher glycogen content when compared with AL flies. Thus, the results of the present study show that at the last tested generation, Assay 2 PR flies' glycogen content is much like that of AL flies. Taken together, it suggests that PR has almost consistently decreased the glycogen levels in flies, thereby showing that this sugar reserve can be drastically influenced by protein in the diet.

## 4. Discussion

In the last four decades, the physiological and metabolic state of fruit flies *D. melanogaster* has been an intriguing field of research because of its close association with fly development, morphology and overall energy homeostasis [48,49]. This has enabled researchers to study diabetes, obesity [48,50,51] and other nutrition-related diseases using fruit flies, alongside considering the tight association between organismal fitness and survival. It is also of prime importance to take into consideration the study of energy or storage reserves and their mobilization during stressful conditions such as exercise, starvation and desiccation [48,52,53]. Pieces of evidence show that the flies’ metabolic state of the same generation and also the next generation yielded strong phenotypes like increased SR and growth inhibition [9,17,49,54]. Results of the current study suggest a long-term effect of PR implementation on the resistance to various stressors such as starvation, desiccation, H_2_O_2_-induced oxidative stress, and biochemical parameters like protein content, TGs, free glucose and glycogen content.

The SR of PR flies had increased across generations under long-term implementation (figure 1 *b,c*), except in PRII70 flies probably because the developmental stage of implementation is similar to the AL effect. Interestingly, at gen 15, the reason why PRI70 has lower SR might be due to the TG levels which are similar to AL, while the SR of PRI50 flies was similar to AL despite their higher TG levels than that of AL. The witnessed single-generation effect could have been generation-specific or just a transient observation, even though selection lines for various stressors report increased survival after stress exposure [35]. Our results are in line with that of certain studies suggesting the reduced dietary yeast/protein level increases the SR of flies [12,55,56]. A recent study also reported that the reduction of nutrients in the larval diet can increase the SR of the flies via low insulin signalling, as they undergo adaptations as a precautionary measure for food scarcity in adult life [9].

The SDR of males and females under PR is increased at the tested generations (including gen 45) compared with gen 15 (PRI70 and PRII70; figure 2 *a,b*). These results are partially in line with the results of a study [57] which reported that the long-lived flies have higher desiccation resistance. Despite the lack of differences in TG levels of PRII50, PRII70 and AL, there exist differences in their SDR in gen 15, indicating that the long-term PR diet concentrations can determine the SDR independent of TG levels. PR flies of the current study showed a lifespan similar to that of AL across most generations (S Krittika & P Yadav, unpublished data, 2024) but higher SDR than that of AL, while another study reported an increased desiccation resistance upon a high-protein diet when compared with a high-carbohydrate diet [58]. Thus, here we show that the SR and SDR of PR flies across generations were higher than those of AL flies. More importantly, it increased across generations when compared with its counterparts of the tested earlier generations and would be one of the important parameters to assess an optimum PR diet.

Our results of higher oxidative stress resistance in PR flies (PRI70) at gen 50, in males (PRII50, PRI70, PRII70) and females (PRI50, PRI70, PRII70; figure 3) are contrary to the study [56], which reported that the DR had no effect (in the early age of the flies) or negative effects (at the later age). Although we did not statistically compare the sex differences under PR diets, it was reported elsewhere that under AL and PR, males have higher stress resistance than females [59] wherein the compensatory feeding of the flies is claimed for increased intake of paraquat, thereby no effect upon DR is observed. As discussed earlier, this could be studied further to understand its correlation with an unaltered lifespan across most generations (S Krittika & P Yadav, unpublished data, 2024), while some studies report a correlation between a longer lifespan and increased resistance to oxidative stress, supporting the free radical theory of ageing [60]. In flies, the methuselah mutants are reported to have an increased lifespan and resistance to oxidative stress [25]. Thus, the long-term PR implementation rendered an increased resistance against oxidative stress and need not be always associated with a higher lifespan.

Our study shows that the long-term PR implementation has increased the protein content in the fly body when fed with a PR diet from the pre-adult or adult stage (figure 4 *a–d*). This is in line with a study conducted with amino acid depletion that showed reduced protein content in emerging adult flies [61] and higher protein levels in pupating larvae of medfly *Ceratitis capitata.* Similarly, another study reported the consistently high protein content of flies across ages that were fed with a full diet, which decreased drastically once fed with a diet of sucrose only [62]. This suggests that the consistent variations in protein content observed across studies may indicate a fundamental biological response to dietary changes or nutrient availability in flies. In the case of lipid content, the current study suggests that the TG content of PR flies was higher than that of AL, while reversal to AL has dropped the TG content of PR flies similar to the level of AL (figure 5 *a–d*). Interestingly, Kristensen *et al*. report increased relative lipid content but lower pre-adult survivorship upon protein-enriched diet for generations [63], which was not the case with the PR diet implemented over generations (S Krittika & P Yadav, unpublished data, 2024). Our results showing increased TG content upon feeding a low-protein diet are consistent with other earlier studies [9,64]. Similarly, our results of higher SR and TG content (even though the correlation is not universal) are in line with other studies [40,65], while a higher lipid level can be induced by a low-protein diet [36,55,64]. Collectively, our findings suggest that yeast content can be a costly dietary component for flies and may play a significant role in fly metabolism [61,62]. However, further investigation is warranted to elucidate the underlying mechanisms that drive these observed effects.

The sugar reserve of glycogen (needed for survival under a diet-restricted environment; [48,66]) and free glucose were assessed in the PR flies (figure 6 *a–d*). In an earlier study, it was reported that glycogen levels decreased upon a low-carbohydrate diet [8], and probably the effect was a direct influence of a carbohydrate diet on the sugar reserve of glycogen. However, we found that the free glucose content of PR flies is lower than that of AL flies upon reversal, the PR males showed higher glycogen content (figure 7 *a–d*). We also observed a sex-specific effect of PR on glycogen reserves probably due to the protein-dependent metabolism and energy utilization variations during reproduction in females. The glycogen level results from our study exhibited differences with the implemented larval PR diet, contradicting the findings from the study by Rehman & Varghese [9]. This suggests that modifying any major nutrient in the fly diet can influence storage reserve levels. Long-term PR resulted in increased stress resistance, protein and TG levels while having no significant effect on glycogen levels. Taken together, biochemical parameters indicate that the observed outcomes are under a transient effect of nutrition available at the developmental stage and only long-term PR can keep the tested parameters at a high level, while reversal to an AL food might reduce the witnessed storage reserve content.

## Data Availability

All data needed to evaluate the conclusions in the paper are presented in the paper. Supplementary material is available online [67].
